# Long-distance migrations and seasonal movements of meagre (*Argyrosomus regius)*, a large coastal predator, along the Iberian Peninsula coast

**DOI:** 10.1186/s40462-024-00469-7

**Published:** 2024-05-09

**Authors:** Miguel Gandra, Alexander C. Winkler, Pedro Afonso, David Abecasis

**Affiliations:** 1 Centre of Marine Sciences (CCMAR), University of the Algarve, Faro, Portugal; 2 Department of Ichthyology and Fisheries Science, Rhodes University, Makhanda, South Africa; 3 Institute of Marine Sciences - OKEANOS, University of the Azores, Horta, Portugal; 4 Institute of Marine Research - IMAR, University of the Azores, Horta, Portugal

**Keywords:** Movement ecology, Pop-up satellite archival tags, Acoustic telemetry, Predatory fish, Migration, Philopatry

## Abstract

**Background:**

The meagre, *Argyrosomus regius*, is a large coastal predatory fish inhabiting waters from the north-eastern Atlantic and Mediterranean Sea, where it is targeted by commercial and recreational fisheries. Previous genetic studies have found an unexpectedly high population differentiation not only between the Atlantic and the Mediterranean, but also along the Atlantic coast. However, the reasons underpinning this genetic barrier remained unclear. Likewise, even though the species is amongst the world’s largest marine teleosts, knowledge about its movement ecology and migratory behaviour remains notably scarce, and primarily reliant on fisheries-dependent data.

**Methods:**

In this study, we used a combination of acoustic telemetry and pop-up satellite archival tags to investigate the movements of 22 adult meagre (70–143 cm total length) along the Southwestern coast of the Iberian Peninsula.

**Results:**

Our results strongly suggest that the previously reported genetic differentiation is not maintained by limited adult dispersal/movement, as hypothesized. On the contrary, we documented some of the longest individual annual migrations ever recorded for a coastal teleost, up to > 2000 km, with frequent back-and-forth movements between the West and Southern Iberian coasts. Moreover, their detected regional movement patterns support the existence of a marked seasonal behavioural shift, with individuals being less active and moving to deeper waters during winter, and are consistent with spawning philopatry associated to their summer reproductive movements. Finally, we identified putative aggregation areas that may harbour important feeding/overwintering grounds.

**Conclusions:**

These findings shed new light on the movement and behaviour patterns of meagre that may be of particular importance for the conservation and spatial management of this species throughout its range, and open the door to further research on functional connectivity.

**Supplementary Information:**

The online version contains supplementary material available at 10.1186/s40462-024-00469-7.

## Introduction

Knowledge about species movements is of great importance to fisheries science and spatial management planning [1–3]. The way aquatic animals move across their seascape and the mechanisms underpinning individual habitat use determine the probability of interaction with anthropogenic activities and can, ultimately, regulate species’ resilience to on-going ocean impacts. Moreover, their distribution and space-use patterns are strongly linked to numerous behavioural, physiological and genetic traits [4, 5], including, for example, trophic relationships in community assemblages [6] and the degree of genetic connectivity between different populations [7], which, in turn, have important evolutionary and fisheries management implications.

A number of recent technological and analytical advances have led to a rapid ascent of animal-borne telemetry in research [8]. This enables scientists to overcome some of the challenges associated with underwater tracking and allow us to “follow” the animals on their natural environment, as they move across their habitat on multiple spatial and temporal scales. As a consequence, movement ecology has been increasingly integrated in informing decision making and improving current management planning [1]. Traditional approaches such as marine protected areas (MPAs), for instance, can be more effectively implemented if critical habitat and individual home range sizes of target species are known [9, 10]. By identifying particularly sensitive areas, such as feeding grounds, nursery habitats [11] and/or aggregation sites [12], as well as the degree of spatiotemporal overlaps with identified threats (e.g., fishing, [13], marine traffic, [14]), essential fish habitats (EFHs) can be prioritized in spatial management planning, minimizing impacts on threatened species. Moreover, knowledge of the areas and migratory routes traversed by highly mobile species can be used to ensure the preservation of habitat connectivity and gene flow [15], through the creation of protected corridors and protection networks [16, 17].

The resilience of meso and apex predators is particularly important due to their ecological role and potential for originating top-down control disruptions, which may cause unpredictable cascading effects across trophic webs [2, 18, 19]. Given their characteristic K-selected life history traits (i.e., slow growth, late maturity and low fecundity), these species are typically more vulnerable to anthropogenic impacts, and substantial population declines. Two of the largest sciaenids (*Totoaba macdonaldi* and *Bahaba taipingensis*), for example, were almost driven to extinction by a combination of overfishing and environmental changes [20, 21].

Another sciaenid, the meagre, *Argyrosomus regius* [22], is a large coastal predatory fish inhabiting the northeastern Atlantic and Mediterranean Sea, where it is one of the most targeted species in small-scale commercial and recreational fisheries. It is one of the world’s largest marine teleost fish, with anecdotal reports of individuals reaching up to 230 cm in length and weighing up to 100 Kg [23]. According to available literature, the estimated length at first maturity in the northeastern Atlantic ranges between 47 cm and 100 cm for females, and is around 62 cm for males [24]. They can be found on shelf waters up to 300 m deep [25] but occur more commonly between 5 and 75 m [26], usually close to the bottom. Adults feed on a large range of prey, ranging from clupeiformes to demersal fish and cephalopods [27]. Spawning is thought to occur around or within river estuaries, where they aggregate in large numbers mostly during summer months and, to date, six main spawning grounds have been identified: the Gironde (France), Tejo (Portugal) and Guadalquivir (Spain) estuaries in the European coast, the Lévrier Bay / Banc D’Arguin (Mauritania) in northwestern Africa; the Nile (Egypt) and Menderes (Turkey) deltas in the Mediterranean [28].

Previous genetic studies have unveiled an unexpectedly high degree of population differentiation not only between the Atlantic and Mediterranean, but also between the West and South coasts of the Iberian Peninsula [28, 29]. More recently, Abecasis et al. [30] confirmed this populational subdivision through a multi-disciplinary approach, combining DArT sequencing and mitochondrial DNA analysis with biotelemetry and biophysical models. With some of these populations being separated by less than 200 km, one of the hypotheses put forth was the existence of potential geographic or oceanographic barriers to dispersal, such as the Cape Sagres, which marks the south-westernmost point of continental Europe and comprises a biogeographical transition area between temperate and subtropical waters [31]. Yet, the spatial behaviour and migratory movements of meagre remain poorly documented, and not at all in some regions, as do the ecological mechanisms driving this genetic differentiation.

In the present study, we used a combination of pop-up satellite archival tags and acoustic telemetry to investigate the movements of adult meagre along the Iberian Peninsula coast. Specifically, our objectives were to: (I) describe their horizontal movements along the continental shelf, (II) identify potential aggregation sites, (III) quantify behavioural changes associated with diel/seasonal cycles, and finally (IV) to assess the level of putative spawning philopatry (interannual fidelity to breeding grounds).

## Materials and methods

### Acoustic telemetry

A total of 22 adult individuals (ranging from 70 to 143 cm total length TL) were captured between 2018 and 2020 (Table S1) and tagged with acoustic transmitters (Vemco© V16-5x model; 162 dB power output, 60 to 120 s nominal delay and expected lifetime of 1292 days). All individuals were caught in an offshore tuna trap in Algarve, the south coast of Portugal (“Tunipex”, Olhão), except two that were tagged in the Tejo estuary (Table 1) using rod and line. Transmitters were implanted internally in the abdominal cavity by placing the individuals in an inverted position on a soft stretcher while providing them with a continuous seawater flow through their mouth and gills, and making a small incision on their ventral region. After the insertion of the acoustic tags, the incision was closed using absorbable sutures (BBraun, Novosyn).

**Table 1:** Summary data for meagre tagged with acoustic transmitters. Detection span: days between release and last detection. Nº Receivers - number of receivers in which each individual was detected; I_R_ –residency index. Mean values are displayed on the bottom (± SE). IDs with an asterisk were double-tagged with acoustic and satellite (PSATs) transmitters. APPA: offshore tuna trap (Olhão, Algarve)

ID	Transmitter	Length (cm)	Tagging location	Tagging date	Last detection	Nº Detections	Nº Receivers	Detection span (d)	N days detected	I_R_
#01*	A69-1602-23797	131	APPA	20/09/2018	23/11/2019	1757	9	430	28	0.07
#02*	A69-1602-23798	128	APPA	20/09/2018	22/11/2021	289	13	1160	15	0.01
#03*	A69-1602-23800	131	APPA	20/09/2018	30/09/2021	2240	51	1107	33	0.03
#04*	A69-1602-23799	132	APPA	20/09/2018	-	-	-	-	-	-
#05*	A69-1602-23802	142	APPA	20/09/2018	04/01/2022	4093	35	1203	83	0.07
#06*	A69-1602-23801	127	APPA	20/09/2018	23/08/2020	3715	60	704	70	0.10
#07	A69-1602-23836	126	APPA	09/07/2019	02/07/2022	977	12	1090	27	0.02
#08*	A69-1602-23834	122	APPA	09/07/2019	08/07/2020	515	22	366	14	0.04
#09	A69-1602-23835	135	APPA	09/07/2019	15/11/2019	887	34	130	24	0.18
#10	A69-1602-23833	124	APPA	09/07/2019	06/07/2022	862	28	1094	32	0.03
#11	A69-1602-23799	125	APPA	10/07/2019	23/07/2020	323	9	380	10	0.03
#12	A69-1602-23803	134	APPA	10/07/2019	18/02/2020	314	15	224	10	0.04
#13	A69-1602-23804	134	APPA	10/07/2019	12/07/2022	2629	41	1099	27	0.02
#14	A69-1602-23805	134	APPA	10/07/2019	12/10/2022	398	3	1191	30	0.03
#15	A69-1602-23806	128	APPA	10/07/2019	27/08/2019	236	4	49	9	0.18
#16	A69-1602-23827	100	Tejo	22/07/2019	27/08/2021	13,314	27	768	287	0.37
#17*	A69-1602-23828	143	APPA	27/09/2019	29/10/2019	54	5	33	2	0.06
#18*	A69-1602-23829	131	APPA	27/09/2019	16/07/2021	186	9	659	13	0.02
#19*	A69-1602-23831	112	APPA	27/09/2019	09/06/2022	238	3	987	4	0.00
#20*	A69-1602-23832	126	APPA	27/09/2019	16/07/2021	110	4	659	7	0.01
#21*	A69-1602-23830	130	APPA	27/09/2019	18/07/2020	143	2	296	3	0.01
#22	A69-1602-23808	70	Tejo	28/05/2020	18/12/2021	46,805	12	570	443	0.78
Mean	-	126 ± 3	-	-	-	3814 ± 2138	19 ± 4	676 ± 85	56 ± 22	0.10 ± 0.04

**Fig. 1:**
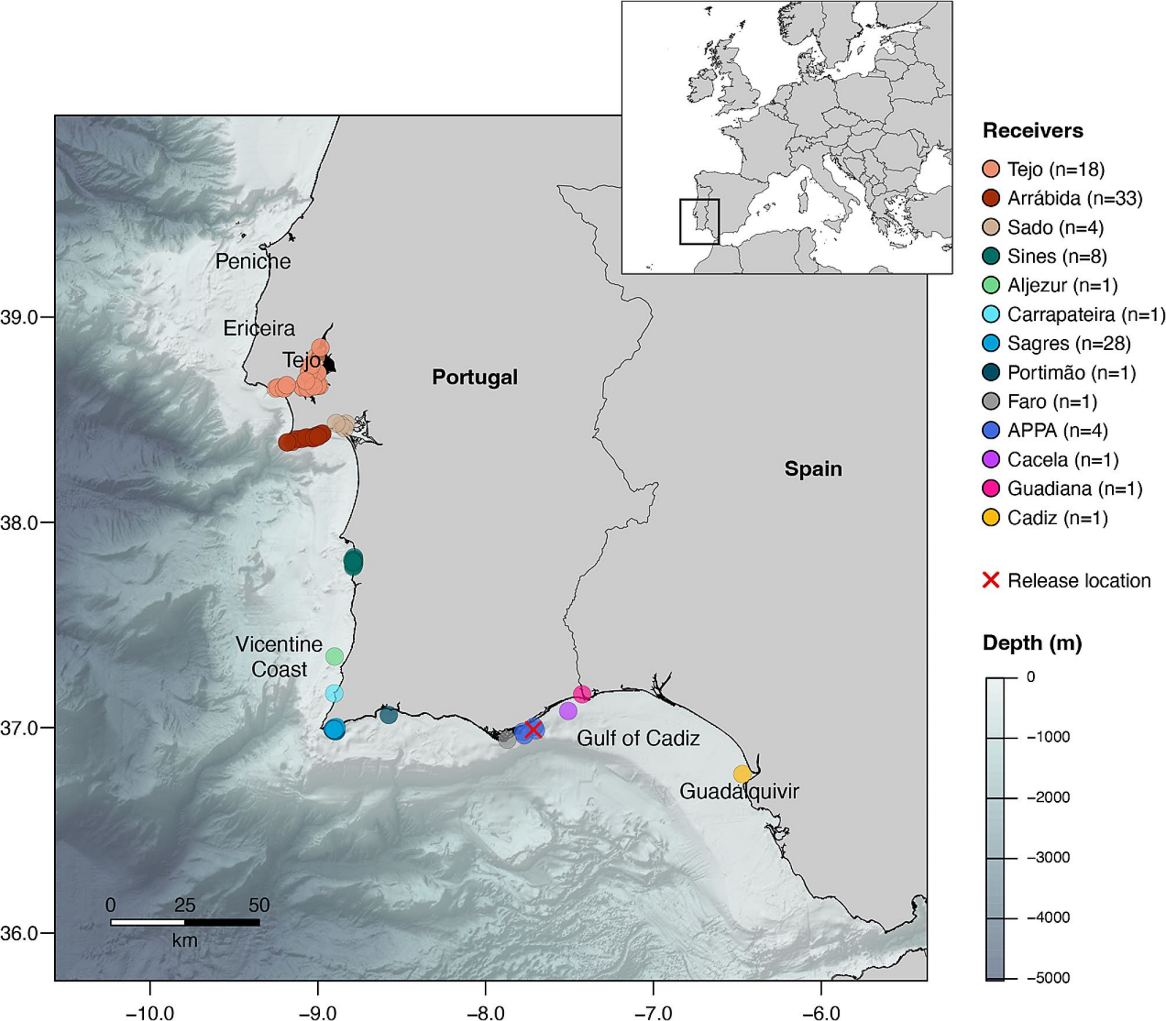
Study area (SW Iberian Peninsula, Europe) and locations of the deployed acoustic receivers (color-coded by site). Nº of receivers deployed on each site is indicated in parentheses. Release location of all specimens except two is indicated by a red cross. The remaining two individuals were tagged in the Tejo estuary. Bathymetric data illustrates the average depth below sea level

To monitor the presence of the tagged meagre, an acoustic receiver array was deployed throughout the Algarve region, including two receivers in the Guadiana estuary, three receivers around the offshore tuna trap in Olhão (APPA), one in the main Ria Formosa entrance, and one offshore Portimão (Fig. 1). Additionally, this study benefited from collaborations with other ongoing telemetry projects which significantly broadened the extent of the monitored area, with 18 receivers deployed in the Tejo estuary, 37 receivers deployed within the Arrábida MPA and the nearby Sado estuary, and 38 receivers in the Vicentine coast (Sines, Aljezur, Carrapateira and Sagres; Fig. 1). Preliminary tag performance tests showed detection ranges of approximately 800 m.

### Pop-up satellite archival tags (PSATs)

To fully understand the migration dynamics of meagre at broader geographical scales, 13 of the captured individuals were double-tagged with pop-up archival satellite transmitters (MiniPATs; Wildlife Computers, Redmond, WA, USA). These tags (124 mm length, ~ 60 g total mass) were attached via a titanium anchor darted into the fish dorsal musculature and between the pterygiophores, and were programmed to record temperature, pressure (depth), 3-axis acceleration and light levels every 3–5 s, depending on the deployment period (varying from 120, 180 and 300 days; Table 2). All data apart from acceleration were internally summarised for transmission. Temperature was measured with a resolution of 0.05º C (sensor range between − 40º C and 60º C), depth with a resolution of 0.5 m (sensor range from 0 to 1700 m) and acceleration with a range of ± 2 g. At the end of the pre-set period, the pin in the anchor starts to corrode, releasing the tag from the animal and allowing it to surface. When at the surface, it begins to transmit data summaries via Argos satellites and to emit short ping signals (similarly to a VHF tracking transmitter). By using its satellite location and this homing pinger, physical recovery was attempted whenever possible in order to obtain the full archival dataset. To avoid losing tags due to premature death, tags were programmed to release whenever depth readings were constant for a period of 5 days.

### Data analyses

#### Passive acoustic telemetry

Acoustic detections were compiled from the ETN database [32] and all data processing and statistical analyses were conducted using the R programming environment [33]. First, spurious acoustic detections (i.e., occurring isolated in periods < 24 h) were discarded to prevent false-positive errors. The remaining detections were then plotted over time to illustrate individual patterns across different locations and reproductive seasons. An overall residency index (IR) was calculated for each individual, by dividing the number of days a fish was detected (D_D_) by the number of days between its release and last detection (i.e., total period; T_P_). This index ranges from 0 (no residency) to 1 (full-time resident), representing the percentage of time at liberty that each fish spent within the monitored areas [34].

Detections were then pooled into 60-min bins and classified according to time of day, month and reproductive season. Time of day (diel phase; day vs. night) was defined based on daily sunrise and sunset times estimated for the study area, using the “maptools” package [35]. Reproductive seasons (resting/spawning) were assigned based on available literature, assuming a spawning period from March to August, similar to that reported for the Gulf of Cadiz [24].

To assess the existence of potential aggregation events, we estimated co-occurrences based on the time and location of the acoustic detections, assuming that joint space usage occurred whenever the individuals overlapped in space (receiver) and time (bin), as detailed in Gandra et al. [12]. To evaluate the timing and periodicity of these possible aggregation events, co-occurrence and detections were visually illustrated using color-coded two-dimensional plots.

#### Pop-up satellite archival tags (PSATs)

The trajectories of PSAT-tagged meagre were reconstructed using Hidden Markov models (HMMs) implemented through the HMMoce package [36]. This state-space-based framework consists in comparing the in-situ variables collected by the tags (such as light level, sea surface temperature (SST) and depth) against high-resolution environmental datasets and/or oceanographic models and estimating time-discrete gridded likelihood surfaces. Hence, high likelihood values (close to 1) are assigned to areas where the profiles collected by the tags closely match those predicted by oceanographic models. In order to improve the accuracy of the geolocation process, acoustic detections of tagged individuals were also incorporated (as “known locations”) in the models, by constraining the likelihood distribution of a given day to the respective COA (i.e., centre of activity position, estimated by weighting the mean of the receivers’ locations by the corresponding number of detections over each 24 h time bin; [37]). Similarly, tagging and pop-up locations were used to restrict likelihood distributions corresponding to the first and last deployment days. Given that some of the tags did not transmit their location immediately after surfacing (surface time identified based on depth patterns), we added a buffer around the first transmitted location by multiplying the time difference with a fixed drift speed of 20 km day^− 1^ (based on the average displacement of the tags after detachment, estimated using Argos-transmitted positions). These buffer areas were then converted into a binary likelihood layer or used to mask any other likelihood distributions for the last day, if available.

**Table 2:** Summary data for meagre tagged with satellite transmitters (MiniPATs). Track Distance: total distance based on the reconstructed track (shortest in-water paths); Light, SST, Depth-Temperature Profile (PDT) and Depth columns indicate percent of deployment days with light-based location estimates, sea surface temperature data, depth-temperature profiles and maximum depths, respectively. Observation Likelihoods indicate the data streams used to reconstruct the most probable track for each animal: L - light-based longitude, S - sea surface temperature, H - HYCOM depth-temperature profiles, W - World Ocean Atlas depth-temperature profiles, O - integrated Ocean Heat Content, B - bathymetry. Mean values (± SE) are displayed on the bottom. IDs with an asterisk indicate tags that were physically recovered

ID	Deploy Date	Pop-up Date	Progr Deployment (d)	Deploy Duration (d)	Sampling Freq (s)	Temp Range (ºC)	Max Depth (m)	Track Distance (km)	Light (d - %)	SST (d - %)	PDT (d - %)	Depth (d - %)	Obs. Likelihoods
#01	20/09/2018	18/01/2019	120	120	3	14.3–24.3	125	645	89 (74%)	0 (0%)	36 (30%)	75 (62%)	H B
#02	20/09/2018	19/03/2019	180	180	5	14.3–24.9	82	438	51 (28%)	1 (1%)	28 (15%)	76 (42%)	S O B
#03*	20/09/2018	05/03/2019	180	166	5	13.3–24.8	90	879	145 (87%)	20 (12%)	164 (98%)	167 (100%)	L H B
#04*	20/09/2018	18/10/2018	180	28	5	14.2–24.2	76	172	26 (90%)	1 (3%)	27 (93%)	29 (100%)	L S B
#05*	20/09/2018	08/01/2019	120	110	3	14.5–24.8	64	499	96 (86%)	4 (4%)	93 (84%)	111 (100%)	S O B
#06	20/09/2018	18/01/2019	120	120	3	14.5–24.7	90	418	72 (60%)	2 (2%)	30 (25%)	84 (69%)	S O B
#07	09/07/2019	-	300	-	3	-	-	-	-	-	-	-	-
#08*	09/07/2019	04/05/2020	300	300	3	13.1–24.8	125	2366	251 (83%)	53 (18%)	285 (95%)	301 (100%)	L S B
#17	27/09/2019	29/01/2020	300	124	3	13.7–20.5	80	917	50 (40%)	12 (10%)	71 (57%)	101 (81%)	L S B
#18*	27/09/2019	19/12/2019	300	83	3	13.3–21.8	113	350	71 (85%)	9 (11%)	78 (93%)	84 (100%)	L O B
#19	27/09/2019	-	300	-	3	-	-	-	-	-	-	-	-
#20*	27/09/2019	23/07/2020	300	300	3	13.6–23.4	121	1464	255 (85%)	39 (13%)	294 (98%)	301 (100%)	S O B
#21	27/09/2019	23/07/2020	300	300	3	13.5–22.7	88	833	34 (11%)	14 (5%)	22 (7%)	69 (23%)	S O B
Mean	-	-	-	167 ± 26	-	-	96 ± 6	816 ± 172	-	-	-	-	-

SST likelihood inference was performed using the Multi-scale Ultra-high Resolution (MUR) SST Analysis dataset, temperature-at-depth likelihoods were calculated using the Hybrid Coordinate Ocean Model (HyCOM; [38]) and bathymetry likelihoods were estimated using the European Marine Observation and Data Network bathymetry dataset [39]. Light-based longitude probabilities were calculated using the GPE2 software provided by the tag manufacturer (Wildlife Computers, Inc.). Since the HMMoce package was envisioned primarily for oceanic pelagic species, some adjustments were made to improve its applicability to a semi-demersal species. Instead of estimating the default one-sided depth likelihood (i.e., only excluding areas shallower than the max depth), we assumed that the tagged fish approached the seafloor at least once a day and consequently calculated a two-sided binary layer and discarded areas in the bathymetry map that were not within 10 m of the fish maximum depth at each time step (an approach similar to that described by [40]). Moreover, a maximum seafloor depth of 300 m was defined as a conservative limit for the position estimates whenever maximum depth measurements were not available, based on the maximum depths measured by the deployed PSAT tags (approximately 125 m) and on available literature (15–300 m depth; [25]).

All datasets were interpolated to a grid of 0.03º and a migratory swimming speed of 2 m s^− 1^ (~ 7 km h^− 1^) was used for the model convolution step. This speed was estimated based on empirical knowledge and corroborated by the movement rates observed between different acoustic receivers (Fig. S1). After likelihood estimation, different sets of layers were sequentially combined and passed to a forward-backward algorithm, in order to obtain the posterior distribution for each time point (see 36 and, [41] for further details on the HMM procedures). A single model was then selected for each individual based on Akaike’s Information criterion (AIC) and used to estimate the most probable location for each day. These positions were calculated by averaging the posterior distribution grid at each time point; however, we implemented an additional depth-based correction procedure to optimize model accuracy around complex coastal habitats. This procedure consisted in snapping positions that were not within 10 m of the fish maximum daily depth to the correspondent nearest depth contour, thus ensuring the nonexistence of land overlaps or spurious offshore locations. Finally, temporally-interpolated intermediate positions were added whenever required to calculate shortest in-water paths between each consecutive location (using a least-cost distance approach; “gdistance” package; [42]), and used to estimate minimum travelled distances.

To further investigate changes in behaviour, more specifically those associated with trajectory step length and turning angle, reconstructed tracks were classified into latent behavioural states, using a nonparametric Bayesian framework (mixed-membership method for movement; M4) implemented through the “bayesmove” package [43]. Step lengths and turning angles were estimated, discretized, and used to segment the tracks. Subsequently, these segments were clustered by latent Dirichlet allocation and classified based on two behavioural states: transiting (characterized by large step lengths and small turning angles) and area-restricted (shorter step lengths with lower directional persistence). The number of optimal behavioural states was chosen based on the fewer number of classes that accounted for > 90% of the observations (Fig. S2), following Cullen et al. [43]. Acknowledging the inherent spatial uncertainties and errors associated with geolocation estimates, we conducted a sensitivity analysis by applying the algorithm to various fitted geolocation models and reconstructed tracks. While some variations were observed, overall findings remained consistent, with predominant behavioural patterns persisting across the same broad regions across different model iterations.

As a proxy for overall activity, we calculated the occurrence of high-acceleration events for the retrieved PSATs. Given the low sampling frequency set for the accelerometers (for battery-saving purposes), we adopted an approach similar to that implemented by Wright et al. [44]. First, the magnitude of acceleration of each sample was estimated, by calculating the square root of the sum of squares of each acceleration axis ($MA= \sqrt{{X}^{2}+{Y}^{2}+{Z}^{2} }$). Then, high-activity events were identified based on the 95% percentile of MA values of the individual (i.e., defined as the upper 5% of the MA). Even though this sampling design did not allow to accurately disentangle the static and dynamic components of acceleration and capture all fine-scale burst swimming events, this approach has been shown to be sufficient to capture the overall trend. The frequency of these high-activity events was then examined over different hours and months, and statistically tested through pairwise Wilcoxon signed-rank tests, using Bonferroni correction to adjust p-values and correct for Type I errors. Similarly, daily and seasonal differences in depth were investigated using both archival and transmitted data, calculating distribution densities over different time frames.

#### Seasonal habitat use

To visually evaluate seasonal habitat use and spawning philopatry, we combined all available positions for each individual (reconstructed PSAT tracks and/or acoustic detections) and estimated bivariate kernel utilisation distributions (KUDs), using the “adehabitatHR” package [45]. In order to ensure compatibility and standardize the two data streams (PSAT vs. acoustic), acoustic detections were converted into daily centre of activity positions (COAs; 37), by weighting the mean of the receivers’ locations by the corresponding number of detections over each 24 h period. KUDs were then calculated independently for each fish and season and plotted together with 50% and 95% contours of occurrence probability. In order to simplify the analysis and ensure the accuracy of KUD estimates, only fish monitored in at least two different spawning seasons and having at least 5 daily positions on each period (*n* = 7) were included.

## Results

**Fig. 2:**
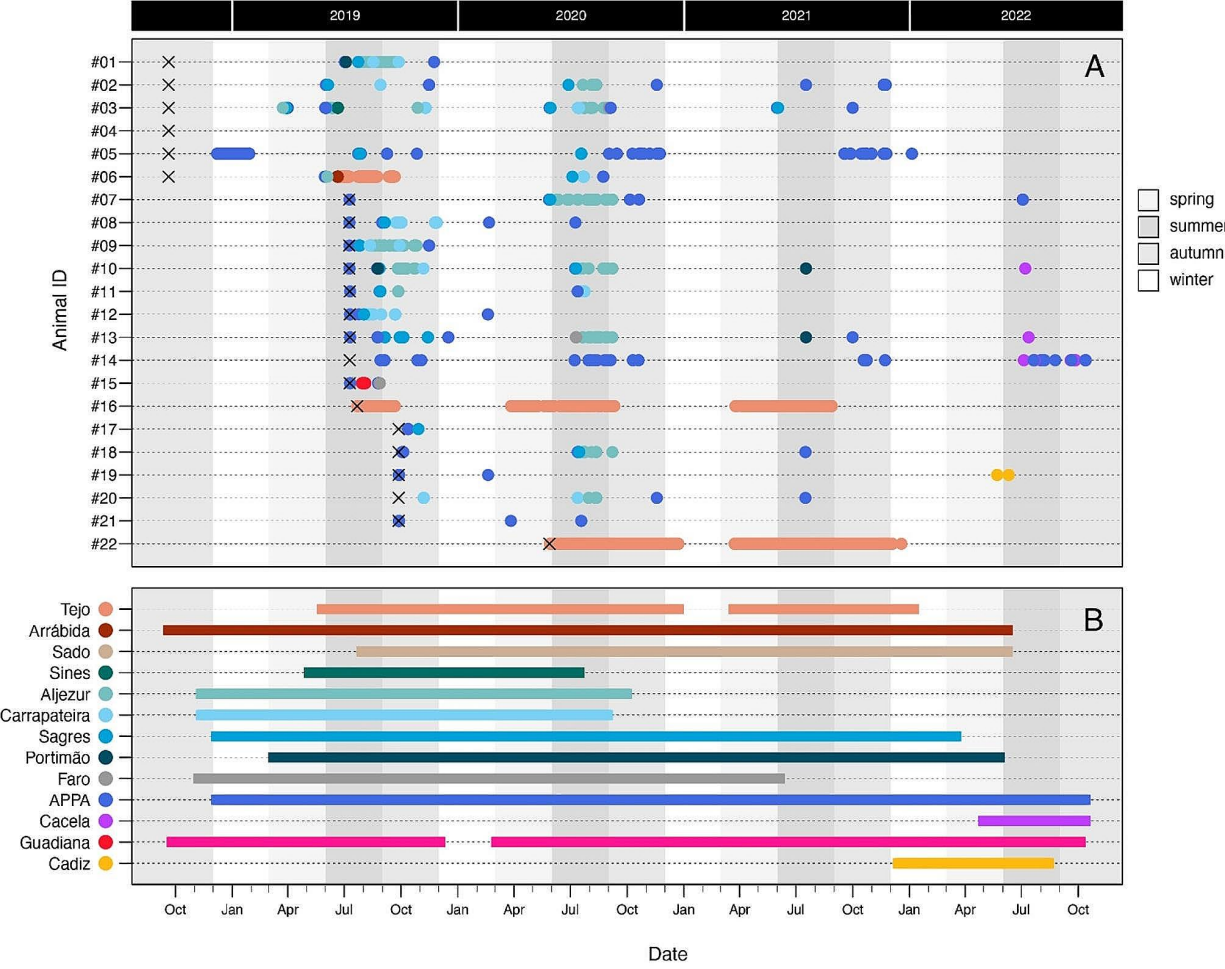
(**A**) Detection patterns of tagged meagre across the study duration, color-coded by site. Cross marks (X) represent release dates. (**B**): Operating periods of acoustic receivers (aggregated by site). Shaded areas indicate different seasons (spring, summer, autumn and winter

**Fig. 3:**
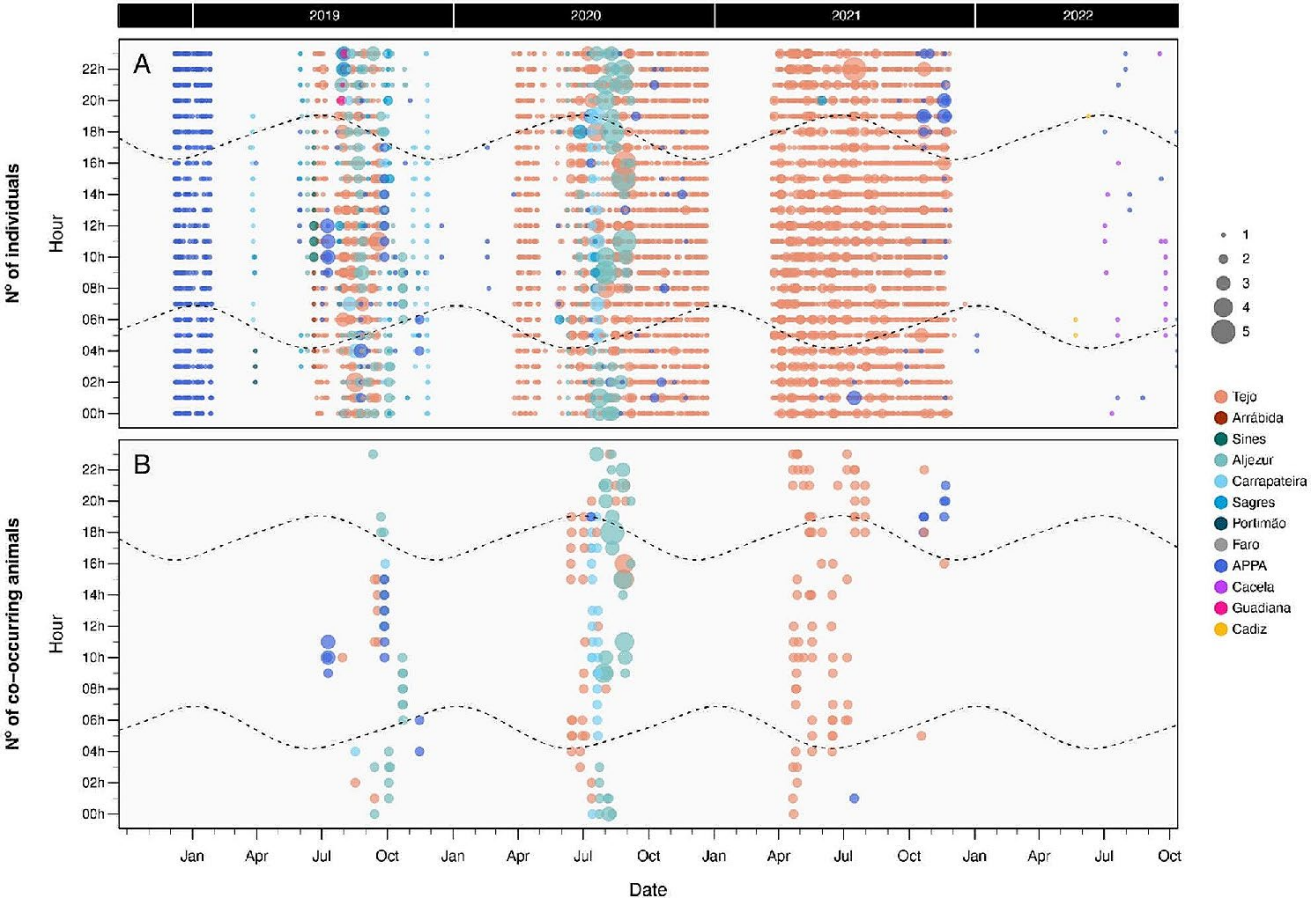
Nº of individuals detected (**A**) and nº of co-occurring individuals (**B**) per hour and date, color-coded by site. Dashed lines correspond to sunrise and sunset periods estimated for the study region, illustrating the annual variation of daylight time

From a total of 22 individuals tagged with acoustic transmitters, only one (#04) was not detected within the receiver array. The remaining fish yielded > 80.000 detections, across individual detection periods ranging from 33 to 1203 days and a mean residency of 0.10 ± 0.04. (Table 1; Fig. 2). Two of the individuals (#16 and #22, both tagged in Tejo) displayed significantly higher residencies, spending respectively 37% and 78% of their detection period within the range of the acoustic network. All detected fish were detected in at least two different receivers, the majority at more than 5 different stations (Table 1; Fig. 2). A significant decrease in detections was observed during winter months (Fig. 2), but no marked differences were found on a diel basis (day vs. night; Fig. 3).

**Fig. 4:**
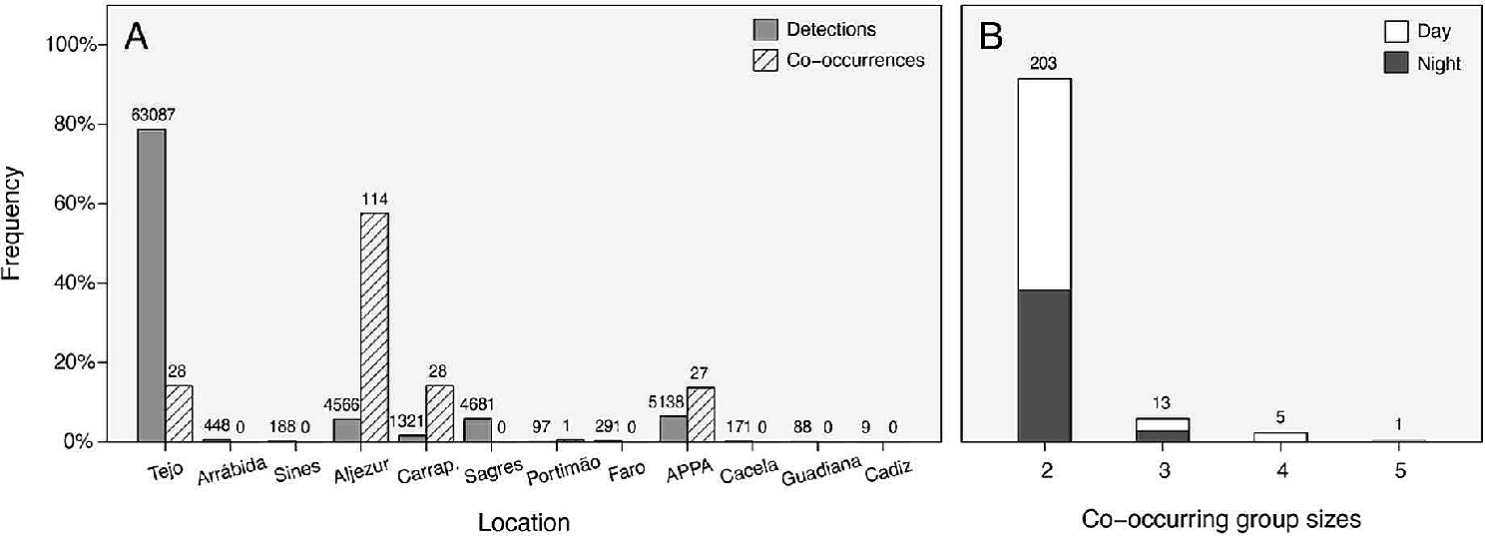
(**A**) Total acoustic detections and co-occurrence frequencies per location. (**B**): Frequency distribution of co-occurring group sizes, divided per diel phase (day vs. night). Absolute counts are indicated above each column

A total of 222 co-occurrence events were registered (Fig. 4A), with > 50% being registered in a single receiver deployed on the Portuguese Southwest coast (Aljezur). Group sizes (i.e., number of fish co-occurring) ranged from 2 up to a maximum of 5 (Fig. 4B), but all co-occurrences involving more than 3 fish occurred on the Aljezur receiver, exclusively during daytime.

From the 13 MiniPATs deployed, 5 relayed summary data through the Argos satellites and 6 were successfully recovered, yielding > 26 million temperature, depth and acceleration readings. Most of the tags (*n* = 8; 62%) remained attached for at least 95% of their programmed deployment duration (Table 2). These deployments lasted for an average of 167 days, ranging between 28 and 300 days. Fish #04 was only monitored for 28 days since it was recaptured in the same tuna trap after one month. Similarly, fish #08 was also recaptured at the tagging location, but only one year later and after its tag had popped up. Neither fish showed any evidence of negative effects associated with tag attachment. Out of the 11 tags that provided data (via satellite uplink or through physical recovery), the percentage of environmental information that could be used to estimate spatial likelihoods varied significantly (Table 2). Light-based position estimates were available for a median of 83% of the deployment days (range: 11–90%). SST data was available for a median of 5% of the deployment days (range: 0–18%). Data was available for a median of 84% (range: 7–98%) and 100% (range: 23–100%) of the deployment days for depth-temperature and maximum depth profiles, respectively. The maximum depths recorded by tags ranged from 64 m to 125 m, while temperatures varied from 13.1 to 24.9 ºC (Table 2).

**Fig. 5:**
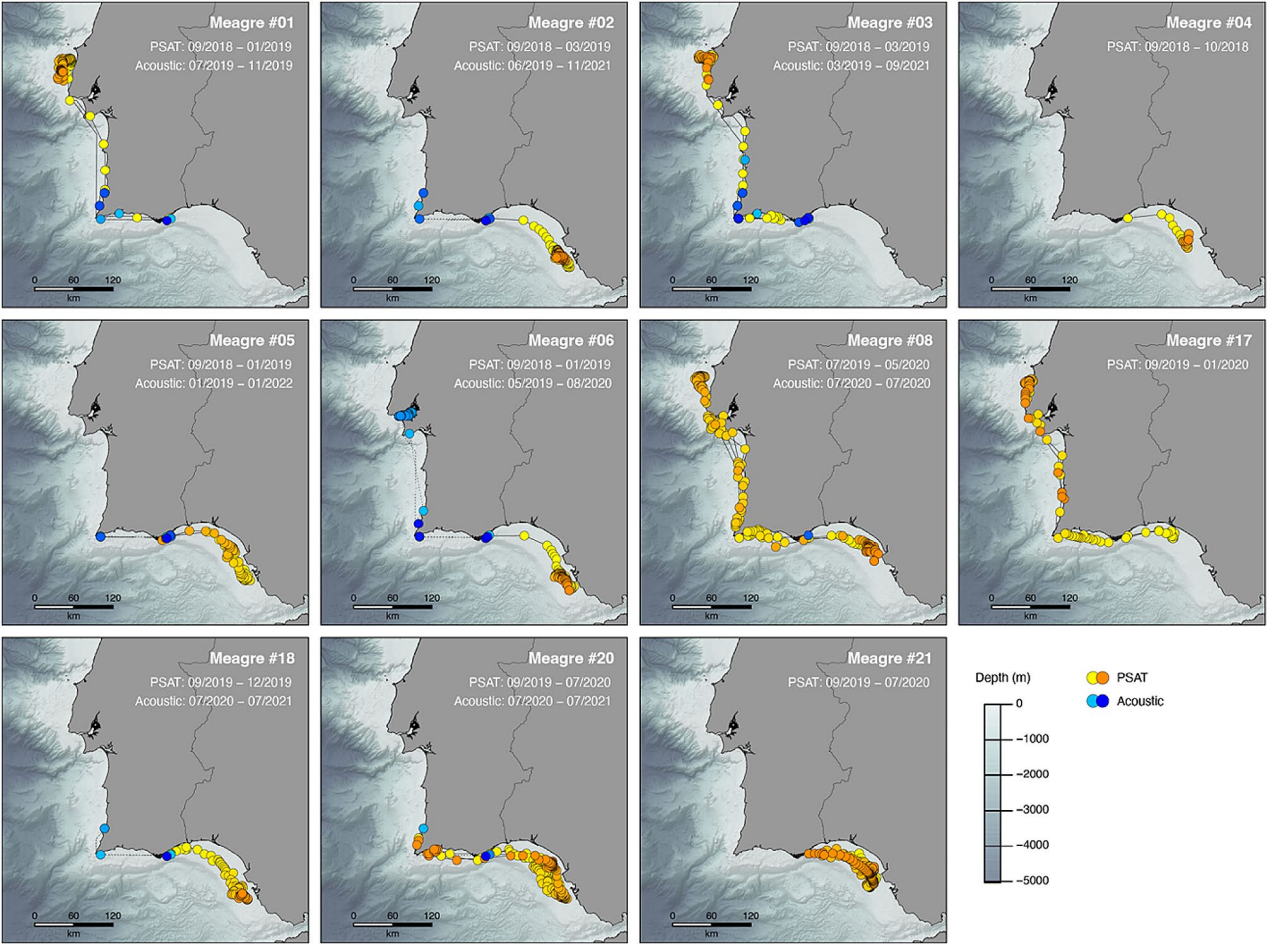
Reconstructed movement trajectories of meagre double tagged with acoustic and pop-up archival satellite tags. PSAT-derived positions are shown from yellow to orange, while acoustic detections are indicated from light blue to dark blue, respectively from the oldest (first position) to the most recent (latest) location. Note: Movements based on acoustic detections are only shown for periods after satellite tag data collection. Background layer illustrates the region’s bathymetry

Reconstructed trajectories of the PSAT-tagged individuals revealed extensive movements between the South and West coasts of Portugal, as well as migrations towards the South of Spain (Fig. 5). Eight out of the 11 tagged fish for which data was retrieved crossed the Sagres cape, four even reaching the Tejo estuary, while nine travelled east towards the Bay of Cadiz and the Guadalquivir estuary, one of meagre’s major spawning areas. Six out of the 13 individuals travelled more than 500 km, with one fish covering more than 2300 km in less than a year (Table 2 and Fig. S3).

**Fig. 6:**
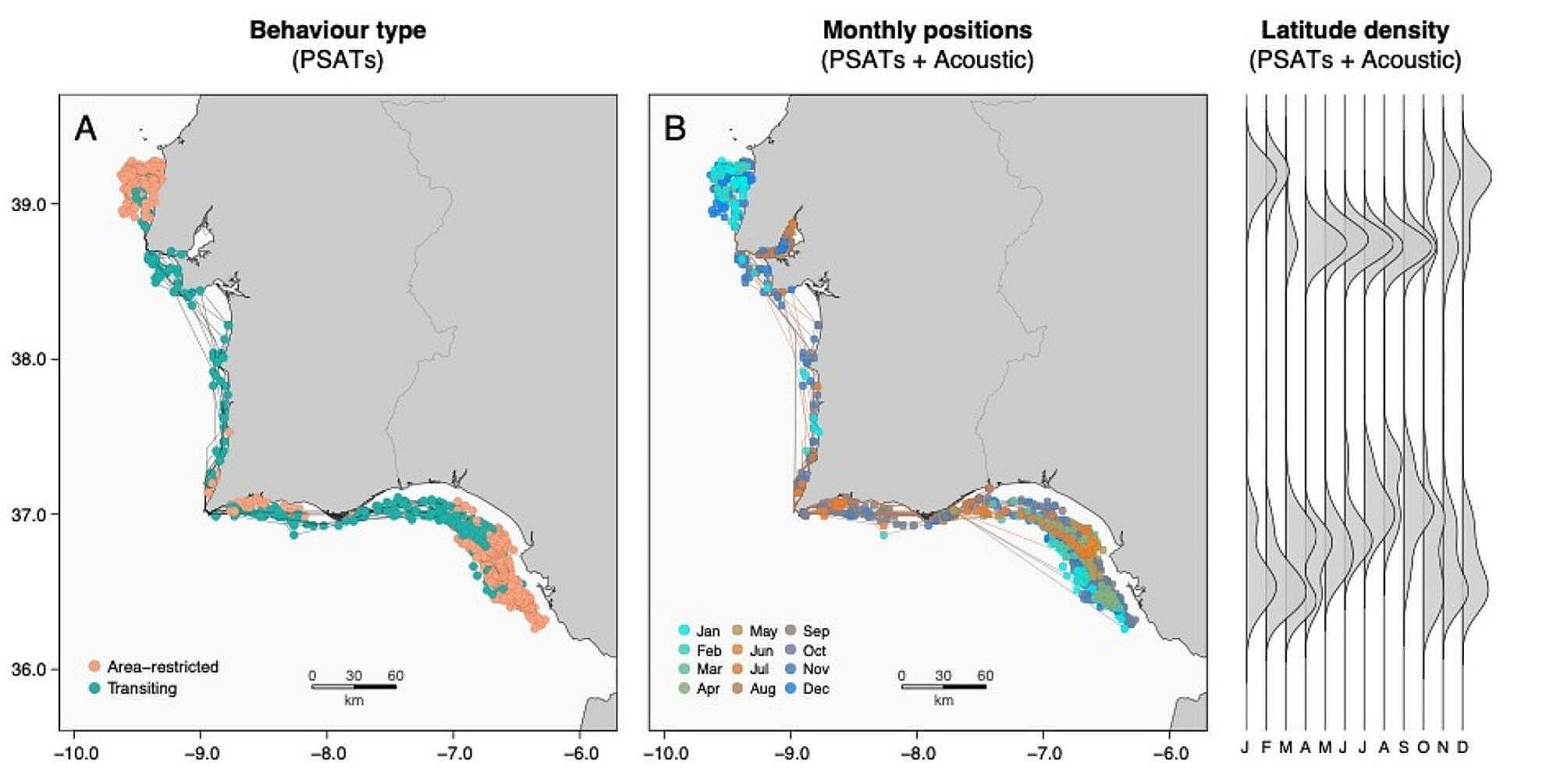
(**A**) Results of the behavioural segmentation analyses, showing all trajectories of the satellite-tagged fish coloured by behaviour type (area-restricted vs. transiting, based on step lengths and turning angles). (**B**) Map with all positions combined (including those inferred from PSATs and those resulting from acoustic detections), coloured by month. (**C**) Monthly latitude densities of all fish positions. Letters below indicate each month (from January to December)

**Fig. 7:**
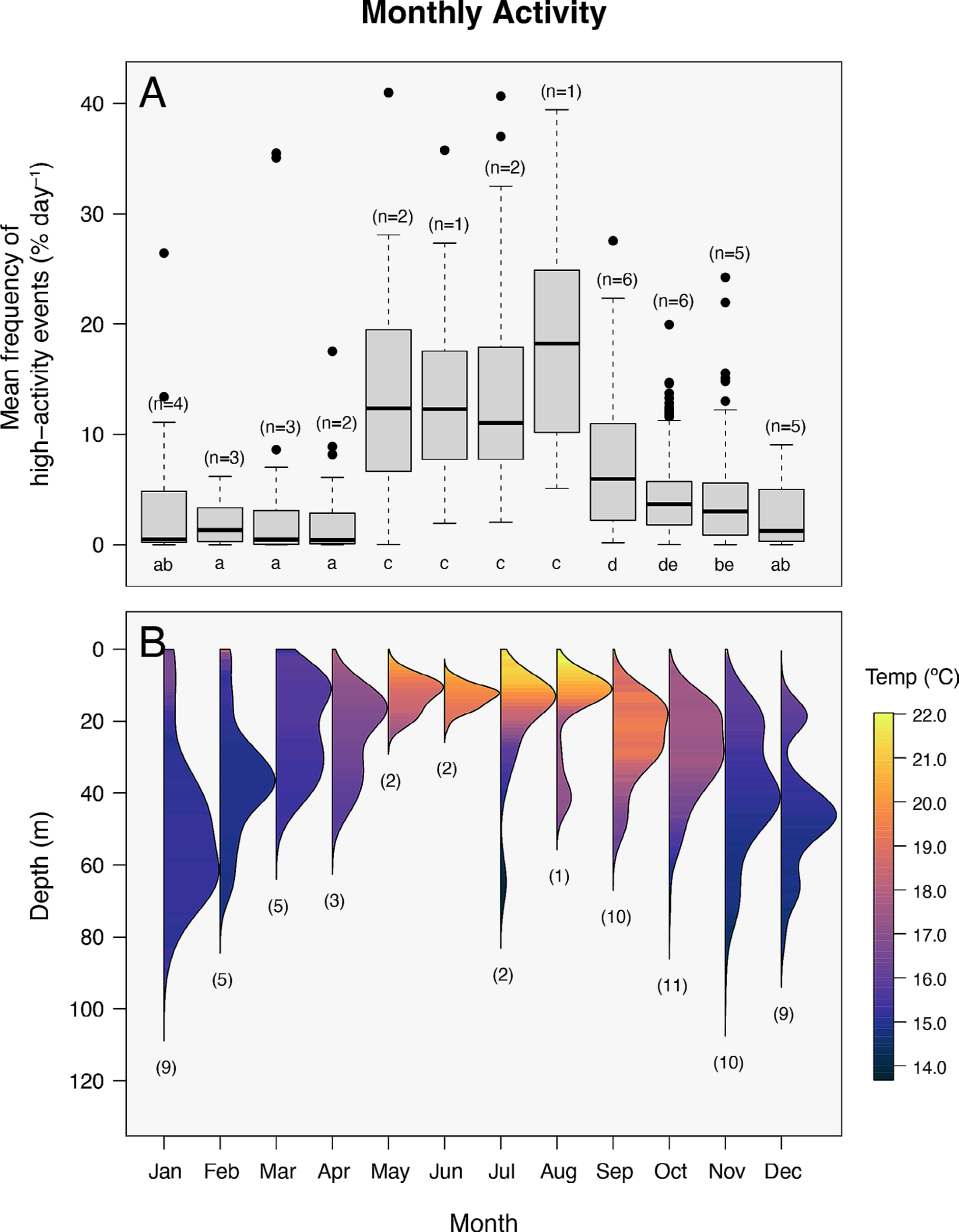
(**A**) Mean percentage of high-activity events (% day^−1^) per month. Boxes’ upper and lower limits represent 75th and 25th quartiles, horizontal lines represent medians, whiskers represent values within 1.5 interquartile ranges and dots indicate outliers. Number of individuals with high-resolution archival data available for each month is indicated above each box plot. Lowercase letters below the boxes represent significance groupings after a pairwise comparison (using Bonferroni correction) where groups sharing the same letter are not significantly different at *p* < 0.05. (**B**) Density of depth usage by month, color-coded by average temperature (1-m depth bins). Number of individuals with depth data (either transmitted or archival) for each month is indicated below

Three potential aggregation areas were identified by the behavioural segmentation algorithm (Fig. 6A). Individuals spent more time and exhibited an “area-restricted” type of behaviour in the western offshore waters northward from the Tejo estuary (between Ericeira and Peniche), in the southwestern Portuguese coast (Sudoeste Alentejano and Vicentina Coast Natural Park), and in the southern Spanish coast around the Gulf of Cadiz. Seasonal patterns were also clearly discernible in movement and behavioural patterns, with individuals moving more offshore and becoming less active during colder months (Figs. 6B and 7A). This finding was supported by the acceleration and depth occupation estimates, with individuals occupying shallower waters and showing a higher percentage of high-activity events during summer months (Fig. 7). Comparatively, no significant behavioural fluctuations were found on a diel basis (Fig. S4), with only evidence of a small peak in activity being identified during dusk periods.

Regarding spawning philopatry, our results suggest that most of the individuals monitored for more than one spawning season, occupied approximately the same areas during their reproductive months (Fig. S5). Fish #16, monitored for over 760 days, was detected inside the Tejo estuary during spring and summer for three consecutive years, coinciding with the onset of their presumed reproductive period. Only one (fish #06) out of the 7 analysed fish is presumed to have visited different spawning grounds during the monitoring period, being detected inside the Tejo estuary during the 2019 spawning period and moving to the Guadalquivir estuary area in the following year. All the fish tagged in the southern coast eventually returned to this same area, including those that migrated northward during the resting season (#03, #06, #08).

## Discussion

Using a combination of acoustic and pop-up archival satellite telemetry, this study revealed, for the first time, that individual adult meagre perform large migrations and highlights some of their regional connectivity patterns along the Iberian Peninsula. It also sheds new light on the fine-scale behaviour of this iconic species.

### Horizontal movements and seasonal behaviour

Until recently, information on meagre population structure and stock connectivity remained scarce and mostly based on indirect sources such as fisheries landings [24] and otolith geochemical signatures [46]. To date, only three studies have conducted DNA analyses to infer its population structure: Haffray et al. [28] using 11 microsatellites and, more recently, Almeida et al. [29] relying on 15 microsatellite loci and Abecasis et al. [30] using a more powerful set of genome-wide SNP-genotyping and mitochondrial DNA. Although all three studies have reported an unexpectedly high degree of genetic differentiation between meagre populations (not only between the Atlantic and Mediterranean populations, but also within the Atlantic), our large movement and low residency data provide strong evidence against the hypothesis of restricted adult dispersal/movement as a mechanism for genetic isolation, as previously speculated [28]. In fact, to the best of our knowledge, these movements are amongst the longest migrations ever recorded for a coastal species, with an individual travelling more than 2000 km in less than a year. However, not all tagged fish displayed the same wide-ranging behaviour. The two smaller individuals (tagged with acoustic transmitters in Tejo; #16 and #22; 100 cm and 70 cm respectively) were only detected near their site of release accounting for the higher residency, somewhat supporting the hypothesis that immature and pre-adult meagre undergo more restricted dispersal tending to stay in estuarine and shallow coastal waters [47].

Several factors may shape species’ habitat use and migratory patterns. Fish can move between habitats to optimize feeding and reproduction opportunities, as well as to escape unfavourable environmental conditions [48]. By applying a behavioural segmentation algorithm, we identified potential aggregation areas, where individuals appear to spend more time and exhibit an “area-restricted” type behaviour, characterised by smaller step lengths and larger turning angles. At the northmost tip, approximately 1/3 of the satellite-tagged fish (*n* = 4) appeared to concentrate on offshore waters between Ericeira and Peniche during colder months, displaying more convoluted movements. Although no acoustic receivers were deployed in this area, this result is corroborated by the fact that one of the main Portuguese commercial fisheries on meagre takes place precisely in the same region [49]. Indeed, the seasonality in these small-scale fisheries (Central West region) also appears to perfectly match the observed patterns, with a peak in landings in the November-January period [49]. A similar pattern was found in offshore waters at the South Spanish coast, around the Gulf of Cadiz, with up to eight individuals appearing to remain in the area for extended periods. Concomitantly, this region also supports important meagre fisheries, with records of sporadic catches of up to 20 tonnes in a single day [24]. Based on this evidence, we speculate that these regions likely harbour important overwintering grounds for adult meagre. Until now, the location of these habitats remained largely unknown, and were only inferred from occasional reports coming from purse-seine fisheries [49]. Since the propensity to aggregate in specific habitats increases a species vulnerability to overfishing and habitat degradation, ultimately leading to potential stock depletions or even local extirpation [50], these findings could have significant implications for the management and conservation of this species.

Furthermore, as previously suggested [26, 46, 51], our results confirm the existence of a seasonal behavioural shift, with individuals moving to deeper waters and exhibiting less high-activity periods during colder periods. The deployed archival tags yielded the first high-resolution data on the vertical habitat use of adult meagre in the wild and constitute the first direct and fisheries-independent evidence of this change in habitat use (see 26 for further details on the depth and temperature preferences of tagged *A. regius*). These findings match the reduced winter growth and food intake rates observed in farmed specimens [52], and are also in line with the movement patterns reported for the congeneric *A. japonicus* in Australia [53] and in South Africa [54]. While impossible to confirm without dedicated feeding/diet analyses, this striking shift in behaviour and habitat usage may reflect differences in prey availability and abundance, with meagre feeding less intensively and/or targeting different prey types during winter months.

Additionally, some areas within the southwestern Portuguese coast (Sudoeste Alentejano and Vicentina Coast Natural Park) were also found to potentially harbour important habitats for meagre. Remarkably, in addition to the AR-type behaviour evidenced by some of the satellite-tagged individuals, we also registered the co-occurrence of up to 5 individuals with acoustic transmitters in one of the receivers deployed in the area (Aljezur), more than 150 km away from the tagging site. Moreover, some of these co-occurrences included animals tagged in different years. This finding (i.e., simultaneous detection of several tagged fish at a such large distance from the release site in the marine environment) is, to our knowledge, virtually unprecedented in fish biotelemetry studies and may hint the occurrence of large meagre schools in the area. Indeed, this region is known to support a large diversity of marine species due to a combination of a wide habitat diversity and localized upwelling, currently comprising an MPA that extends up to 2 km from the shoreline. Even though some of the satellite-tagged individuals were detected in the area during autumn and winter months (from September to February), most of the acoustic detections and co-occurrence events were registered between July and August, indicating that post-spawning fish may use this area as a feeding ground, and not only as a migrating route towards their overwintering grounds. Given that this region lacks the large estuarine environments typically associated with spawning activity in this species, it is unlikely that these fish are using the area for reproductive purposes.

### Breeding philopatry

Given that our results do not support the hypothesis of limited adult meagre dispersal and exclude the presence of oceanographic barriers as a cause for the observed genetic isolation between western and southern populations, other mechanisms must be driving the high genetic differentiation in meagre. It is now widely accepted that philopatry and homing behaviours can drive significant population structuring in marine fish species, even at scales comparable to those of migrating birds and anadromous fishes [55]. Indeed, this type of seasonal movement strategy has already been described in several sciaenids. Red drum, *Sciaenops ocellatus*, for example, were found to consistently return to nearshore waters off their estuarine nursery for spawning [56]. Similarly, spotted seatrout *Cynoscion nebulosus* were reported to show high fidelity to spawning grounds [57]. Although the sample size and duration of the present study (mainly limited by the battery life of PSATs) preclude us from providing a definitive answer, our results suggest that meagre might exhibit a similar homing behaviour and that this predisposition may be one of the factors responsible for the degree of population structure observed in previous studies. Indeed, from the seven individuals monitored across two or more spawning seasons (PSAT and/or acoustically), six re-used the same areas and exhibited inter-seasonal habitat overlap during reproductive months. Fish #06 was the only one that was detected in two different areas during the spawning period, being detected inside the Tejo estuary in 2019 and in the Guadalquivir area in the subsequent year. This is not surprising considering inter-individual variability and the fact that in many other natal-homing species, not all fish exhibit spawning site fidelity (e.g., mark-recapture studies on Atlantic herring *Clupea harengus* estimate that only between 75% and 95% of the tagged individuals returned to the same spawning grounds; [58]). Simultaneously, it is possible that not all fish within a population conform to the same reproductive cycle, with some of the individuals not spawning every year. Indeed, alternated spawning has been reported at an increasing rate for iteroparous species and is believed to be more widespread than previously assumed [59, 60]. Given that only a fraction of the tagged individuals was successfully monitored across multiple consecutive seasons and that identifying precise spawning grounds of asynchronous batch spawner species is inherently difficult, these assumptions require further testing. Studies conducted on longer (interannual) time scales with more individuals or complementary methodologies (e.g., isotopic and elemental markers) could provide further insights into these important aspects of meagre’s reproductive ecology.

### Fine-scale behaviour

Given that piscivorous, higher trophic species are typically crepuscular or nocturnal foragers [61] and that many small forage fish including clupeiforms such as the european anchovy and sardine (two of the most targeted prey items by adult meagre; [27]) often migrate vertically in the water column [62], one could expect that meagre would follow a similar rhythm and forage more intensively after dark, displaying larger vertical displacements and high acceleration signatures. However, analysis of archival data and acoustic detections revealed minimal variation in depth utilization by tagged individuals throughout the day and no significant differences in the frequency of burst swimming events between day and night periods, except for a slight increase in activity observed at dusk. Together with the lack of discernable patterns in acoustic detections, these results reveal that there is a much more pronounced behavioural shift on a seasonal than on a nychthemeral level. This suggests that as voracious predators with a high diet plasticity and adaptability in foraging (feeding both on pelagic and demersal species; [27, 63]), meagre doesn’t necessarily need to undergo vertical migrations to target specific prey. Instead, it likely relies on a wide range of food sources available throughout the water column, foraging more similarly to cathemeral species, opportunistically feeding throughout the day.

This finding somehow contrasts with the diel behavioural patterns observed in juvenile meagre [47] and those reported for other sciaenids (e.g., *Argyrosomus japonicus*; [64]), which tend to be more active during the night. Yet, the individuals analysed in these previous studies were considerably smaller than the specimens described here. Given the large size of our individuals (mean fork length of 129 cm), it is likely that predation avoidance plays a minor role when compared with the juvenile or sub-adult fish analysed in those studies. Dietary differences are known to exist between juvenile and adult meagre [27], and hence it is likely that a matching ontogenetic shift in diel activity could take place as they begin to target different prey.

## Conclusion

Reconstructed trajectories based on acoustic and PSAT data point towards extensive alongshore movements in the Iberian coast, with some of the tagged fish performing some of the largest migrations ever recorded for a coastal teleost. Hence, we propose that the previously reported genetic subdivision may be explained not by a limited adult dispersal, but rather by a strong spawning site fidelity and philopatric behaviour. Moreover, movement patterns confirm the existence of a marked seasonal behavioural shift, with tagged fish occupying deeper habitats and being less active during the boreal winter. The refinement of geolocation models (comparatively to the commonly used GPE3 software) and application of behavioural segmentation techniques allowed us to identify three potentially important aggregation areas that may serve as overwintering and/or feeding grounds, and that may hold particular relevance for their management and conservation. In conclusion, this study provides valuable new insights into the migratory patterns and habitat use of adult meagre in the Iberian Peninsula and opens the door to further research on this important coastal predator.

### Electronic supplementary material

Below is the link to the electronic supplementary material.

Supplementary Material 1

## Data Availability

Acoustic telemetry data is available at the European Tracking Network data portal (http://www.lifewatch.be/etn/), developed by the Flanders Marine Institute as part of the Flemish contribution to LifeWatch. Satellite telemetry datasets are available on reasonable request to the corresponding author.
