# Supplementary material for: Long-distance migrations and seasonal movements of meagre (Argyrosomus regius), a large coastal predator, along the Iberian Peninsula coast
Source: Mov Ecol. 2024 May 9;12:35. doi: 10.1186/s40462-024-00469-7 (PMC11080147; doi:10.1186/s40462-024-00469-7)
Supplement: Supplementary file 1 — Supplementary Material 1 [file 40462_2024_469_MOESM1_ESM.docx]

**Supplementary Material**

**Table S1.** Summary information for the different tagging batches carried out in the study. APPA: offshore tuna trap (Olhão, Algarve).

|  |  | **2018** |  | **2019** | |  | **2020** | |
| --- | --- | --- | --- | --- | --- | --- | --- | --- |
|  |  | **Sep** |  | **Jul** | **Sep** |  | **May** | **Oct** |
| Tagging location (number of fish caught) |  | APPA (6) |  | APPA (9)  Tejo (1) | APPA (5) |  | Tejo (1) | APPA (8) |
| Acoustic tags deployed |  | 6 |  | 10 | 5 |  | 1 | 8 |
| Pop-up satellite tags deployed |  | 6 |  | 2 | 5 |  | 0 | 0 |
| Number of post-release mortalities |  | 0 |  | 0 | 0 |  | 0 | 0 |
| Average fork length in cm  (± SD) |  | 132 ± 5 |  | 126 ± 10 | 128 ± 11 |  | 70 ± 0 | 132 ± 7 |
| Datasets transmitted by satellite tags |  | 6 |  | 1 | 4 |  | 0 | 0 |
| Number of tags recovered after pop-up |  | 3 |  | 1 | 2 |  | 0 | 0 |
| Average duration of tag attachment in days (± SD) |  | 121 ± 53 |  | 300 | 177 ± 144 |  | - | - |


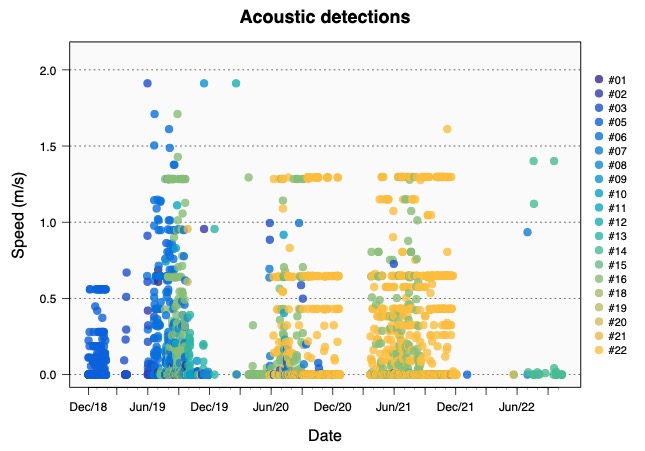
**Figure S1.** Fish mean travel velocity (m/s) inferred from consecutive acoustic detections, color-coded by individual. Distances between different receivers were calculated considering land surfaces (i.e., estimating shortest in-water paths).

**Figure S2.** Proportion of time spent in each behaviour class across all track segments. Two behavioural classes (area-restricted and transiting) were selected as the optimal number of states i.e., the fewer number of state that account for > 90% of the observations, following Cullen, Poli (40).


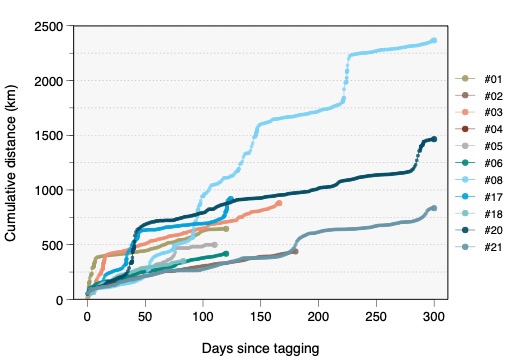
**Figure S3**. Cumulative minimum distances (km) travelled by *Argyrosomus* *regius* tagged with PSATs based on the geolocation models.

**Figure S4.** A: Mean percentage of high-activity events (% day^-1^) per hour. Boxes’ upper and lower limits represent 75th and 25th quartiles, horizontal lines represent medians, whiskers represent values within 1.5 interquartile ranges. Lowercase letters below the boxes represent significance groupings after a pairwise comparison (using Bonferroni correction) where groups sharing the same letter are not significantly different at p < 0.05. B: Density of depth usage by hour, color-coded by average temperature (1-m depth bins). The top horizontal colour bar illustrates the average diel phase corresponding to each hour over the course of 1 year, with blue corresponding to night-times, yellow to daytimes, and orange to crepuscular (dawn and dusk) periods.


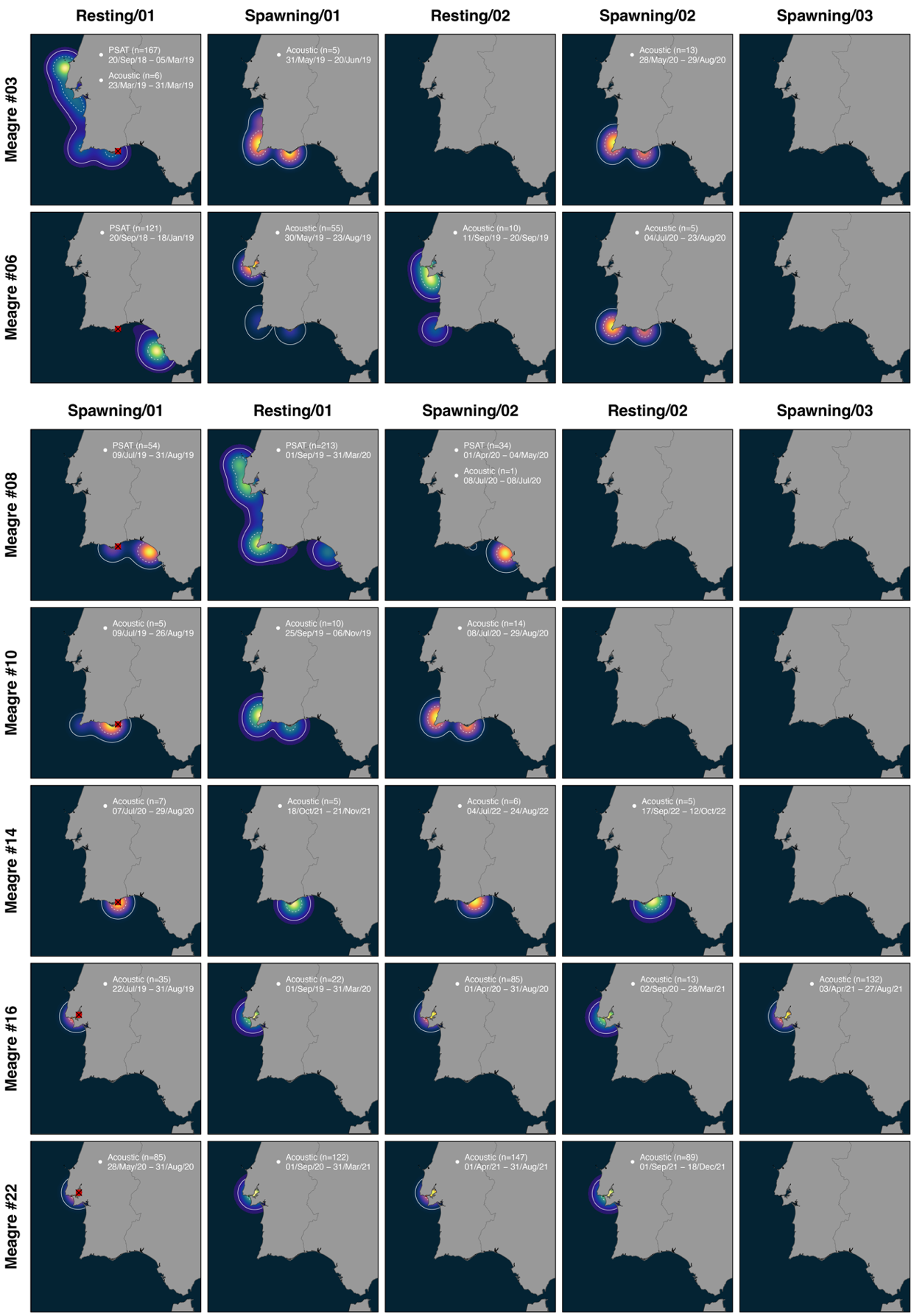
**Figure S5.** Kernel utilization distributions of tagged meagre by reproductive season (including both acoustic and PSAT-derived positions). Only individuals detected in at least two different spawning seasons were included. Dashed lines delimit areas corresponding to 50% of occurrence probability (core-activity areas), while solid lines delimit areas corresponding to 95% of occurrence probability (home-ranges). Red mark illustrates the tagging location and season.
